# Medical News

**Published:** 1855-10

**Authors:** 


### THE MEDICAL EXAMINER.

PHILADELPHIA, OCTOBER, 1855.

### MEDICAL NEWS.

During the week ending September 22d, there were 217 deaths by yellow fever in Norfolk, in a population estimated at 4000. In Portsmouth the deaths amounted to 115 in the same period. The total mortality from the epidemic in Norfolk was estimated at upwards of 2000. It gives us the most heartfelt pleasure to state that there is a considerable decline in the epidemic since the 22d. Very few new cases had occurred, and the number of deaths was much smaller in both places. In New Orleans the epidemic was generally considered at an end, there being but 89 deaths from the disease during the week ending Sept. 22d.

---

The editor of the New Orleans Medical and Surgical Journal makes the following remarks in the September number: "After the most searching investigation, there appears to be an entire unanimity of opinion, both among contagionists and non-contagionists, that the yellow fever of 1855 originated in New Orleans, and that all the earlier as well as the later cases occurred among persons who had not been in any manner exposed to the fever in foreign ports or to imported contagion."

---

Dr. Bulkley has resigned his connection with the New York Medical Times. The great labor and anxiety his position caused him, interfered too much with the discharge of his professional duties. We regret his withdrawal.

---

In the Boston Medical and Surgical Journal of Sept. 20th, there is a short notice of Dr. Turnbull's paper on Hooping Cough, published in the August number of the Examiner. The writer says, "We notice that the author, like many others, writes 'whoop' and 'whooping cough,' 'hoop' and 'hooping cough.' We contend that the former is the orthography, the words *hoop* and *whoop* being in signification as different as they are in spelling."

As this is a very grave matter, we have taken some pains to look into it, and the following is the result of our labors. Webster defines *hooping cough* as a cough in which the patient hoops or whoops with a deep inspiration of breath. On looking for *Whooping Cough* we find it thus—Whooping Cough—see Hooping Cough. The verb Hoop, v. t. is defined to bind, to fasten, &c.; and again: 1, to drive with a shout or outcry—(Shaks.) 2, to call by a shout or hoop. Walker spells it hooping cough. Dr. Dunglison spells it both with and without the W. Drs. Wood, Copland, Watson, Dr. Johnson, in the Cyclopædia of Practical Medicine, Dr. J. F. Meigs, in his work on Diseases of Children, Dr. Williams and Dr. Gerhard, in Tweedie's Library, Dr. Bell, in Drs. Bell and Stokes' Practice, Dr. Walshe, in his "Diseases of Lungs, &c.," and Dr. Forbes, in his Translation of Laennec, all spell it *Hooping* cough.

We do not intend to *hoop* or make a loud cry about the matter; all that we wish to know of our orthographical friend is whether he still holds on to his W. We pause for a reply.

---

Obituary.—Among the many physicians who have fallen victims to the yellow fever in Norfolk, we regret to perceive the name of Dr. Geo. L. Upshur, whose valuable contributions to this journal are well known to its readers. Dr. Upshur was highly respected in Norfolk, where he occupied the post of Surgeon to the Marine Hospital.

